# Editorial overview: Virtual collection on chromatographic and mass spectrometric methods in food, health, and agriculture

**DOI:** 10.1016/j.mex.2022.101628

**Published:** 2022-01-28

**Authors:** Andrew J. Gravelle

**Affiliations:** Department of Food Science and Technology, University of California Davis, Davis, CA, United States

**Keywords:** Dispersive liquid-liquid microextraction, Gel permeation chromatography, GC, GC-MS, HPLC, HPLC-MS/MS, Molecular imprinted solid phase extraction, Solid phase extraction

This virtual collection is focused on the application of chromatographic and mass spectrometric methods being applied in the areas of agriculture, food, and health/nutrition. We preface by noting this is intended as a follow-up to the recent special issue on “Advanced mass spectrometric analysis for environmental and food safety”. Due to the considerable number of protocols in this subject area published in MethodsX to date, the intent of this collection is to augment the previous topic. We hope to highlight additional developments in extraction and quantification techniques, and expand the theme to include several articles which employ analytical techniques to identify and quantify compounds related to health and nutrition. The remainder of this editorial provides a brief summary of the protocols included in the collection, through which we also hope to provide a clear narrative to support this overarching theme.

The identification and quantification of potentially harmful compounds in food and agricultural products is a common application for chromatographic analysis. These techniques can be used to ensure compliance with standards set by regulatory bodies, but updates to such methods are often necessary to reflect current monitoring specifications and advances in analytical instrumentation. Included in this collection are several optimized protocols that have recently been published in MethodsX concerning the detection of pesticide compounds, food dyes, and various small molecule contaminants, both natural and synthetic in origin.

The methodology reported by Melton and Taylor [1] provides an update to their previous protocol for identifying a wide range of pesticide residues and associated metabolites for a variety of fruits and vegetables with both high moisture and high moisture/high acid content. They detail workflows using either gel permeation chromatography (GPC) or dispersive solid phase extraction (d-SPE), which are selected depending on the food matrix. Quantification of pesticides and metabolites was carried out using GC-MS/MS analysis on a triple quadrupole system with cool on-column injection. The methodology also outlines the use of a programmable temperature vaporising injector with a baffled injection linear to improve performance for a subset of pesticides.

Several methodologies have also recently been outlined for detecting regulated compounds and contaminants in meat systems. Extraction of target compounds from meats can be challenging due to the complex solid matrix and presence of interfering compounds. Thus, new and improved methodologies are important for identifying and quantifying relevant compounds in these foodstuffs. The protocol developed by Iammarino et al. [2] details a methodology for detecting 12 food dyes in meat products, intended to ensure compliance with regulatory standards. The method provides a single optimized solvent mixture for the entire suite of dyes, and recommends an ultrasound-assisted extraction procedure. Detection was conducted using an HPLC-UV/DAD (DAD; diode array detector), which demonstrated complete separation of the dyes in samples of fresh pork and beef, as well as salami and seasoned sausage products. This workflow provides a straight-forward sampling procedure capable of unambiguously distinguishing a range of dyes in a single measurement. In addition to additives, potentially harmful compounds can be produced as a result of processing or cooking processes. For example, heterocyclic amines can occur in grilled or charred meats, and may pose a human cancer risk. To address this particular issue, Manful et al. [3] reported a workflow employing a pressurized accelerated solvent exchange method in methanol with a subsequent selective extraction/concentration procedure. Detection of heterocyclic amines extracted from either beef or moose meat was conducted using a UHPLC-MS/MS with high-resolution ion trap mass analyzer. Although numerous protocols identifying heterocyclic amines have been previously reported, this procedure minimizes laborious sample pre-treatments to remove interfering compounds, thus greatly increasing extraction efficiency.

Ochratoxin A is another potential contaminant which can affect meat products. While this mycotoxin most commonly occurs in cereal grains, contaminated feedstocks can lead to bioaccumulation in animal tissues. An extraction protocol was reported by Luci [4] for porcine muscle, kidney, and liver tissues using molecular imprinted solid phase extraction (MISPE) in conjunction with HPLC and fluorescence detection (HPLC-FLD). This method demonstrated a high sensitivity and reproducibility, and the optimized MISPE column conditioning and regeneration procedures allowed for up to 7 repeated uses. A second methodology using MISPE purification coupled with HPLC-FLD was also outlined by Cerkvenik-Flajs and Šturm [5] for the detection of Bisphenol A (BPA) in various edible sheep tissues. BPA is a widely used precursor compound in polycarbonate plastics and epoxy resins, and there are concerns over exposure to free BPA due to its estrogen-mimicking properties. While numerous protocols for the detection of BPA have been published, the use of a single-step MISPE procedure reduces the need for time-consuming and laborious pre-treatments. This article details an optimized SPE procedure from sheep tissue, and reported appropriate column conditioning resulted in no loss of performance with up to 5 cartridge reuse cycles. These two MISPE workflows also demonstrate the potential for Ochratoxin A and BPA quantification in various animal species tissues, and could also be of relevance to the field of veterinary toxicology.

While the aforementioned study provides a method of evaluating indirect exposure to BPA through an environmental route, direct human exposure is most commonly associated with food packaging materials. The methodology of Mahdavianpour et al. [6] outlines a simple dispersive liquid-liquid microextraction (DLLME) pre-treatment which was coupled with HPLC-DAD to determine the content of BPA in a sampling of commercial foods packaged in cans, paper boxes, and glass jars. This procedure provides a rapid, cost-effective concentration protocol for high-throughput sampling, while providing a low limit of detection and high BPA recovery.

Protocols for detecting naturally occurring environmental contaminants have also recently been addressed. The bioaccumulation of arsenic in rice is known to be greater than in other commodity crops, which is particularly relevant due to its broad global consumption. However, the physical form of arsenic impacts its toxicity, as inorganic forms are more acutely toxic than organic species. The method outlined by Urango-Cárdenas et al. [7] adapts a previous microwave-assisted extraction technique used in other food matrices to maintain arsenic speciation. Detection and quantification was conducted using HPLC coupled to a hydride generator with an atomic fluorescence detector (HPLC-HG/AFS) to quantify individual arsenic species in rice grains. Such techniques could be useful for providing a more complete assessment of the potential dietary risks associated with arsenic accumulation in rice throughout various parts of the world.

While chromatographic techniques are valuable tools for detecting regulated compounds and contaminants in foods, they are also regularly used to identify and quantify health-promoting molecules. Several such protocols have recently been published in MethodsX which are included in this article collection. Dell'Aquila [8] outlined a procedure for the extraction and detection of zinc-bound compounds present in produce from the *Brassica oleracea* species (cabbage, broccoli, and kale). While differences in the sample matrix often require varying extraction conditions (even within species), this method succeeded in using a single preparation procedure for all three vegetables. The protocol uses solid phase extraction followed by an additional enzymatic digestion phase prior to concentration. Quantification was achieved using size exclusion chromatography and inductively-coupled plasma mass spectrometry (SEC-ICP-MS). The durability of this method suggests it could have further applications in other foods with complex matrices. Another procedure for evaluating health-promoting compounds was developed by Yonekura and Tamura [9] for quantification of flavanols in the seeds of guarana, a fruiting plant native to the Amazon basin. The protocol greatly reduced analysis time by using isocratic elution, which replaced a lengthy gradient elution process required by previous protocols. The method also demonstrated improved sensitivity, despite using a simple, low-cost HPLC-UV detection system. The method was demonstrated to be suitable for detection of several flavanol compounds in guarana extracts, as well as *in vitro* digesta and Caco-2 cell permeates, which are relevant to bioavailability studies.

Additional procedures for evaluating compounds from food samples subjected to *in vitro* digestion have also recently been reported. Lin et al. [10] developed a methodology which focused on separating and quantifying individual bile salts from digested foods (simulated chyme) by adapting and comparing three previously reported HPLC protocols. The recommended procedure used an ion-pair reagent which allowed for efficient and complete separation and quantification of four primary bile salts. The viability of the method for evaluating food samples was demonstrated using dried beans and major bean components subjected to *in vitro* digestion. Such methods could provide valuable insight into the effect of food matrix on digestion processes. Another recent study by Ogilvie et al. [11] detailed a label-free, quantitative evaluation of immunogenic peptides derived from gluten-containing foods subjected to the INFOGEST simulated digestion assay. The authors outline a quantitative parallel reaction monitoring-mass spectrometry (PRM-MS) method adapted from a previous protocol used to identify peptides from isolated protein systems. This updated procedure outlines the optimization of an appropriate quenching reagent based on the solubility of the proline-rich target peptides. As gluten digestion will be impacted by the food matrix, optimizing the procedure to be compatible with the INFOGEST protocol could provide a means of evaluating effects of processing parameters or formulation changes on the formation of these immunogenic peptides.

Several other protocols recently published in MethodsX are also relevant to the fields of food and agriculture, and are thus included in this collection. Hou et al. [12] developed a procedure to screen for antimicrobial compounds in wastewater produced by a rice spirit distillery. An initial screening procedure led to identification of 5 such compounds in high quantities, which were then simultaneously quantified using UHPLC-MS/MS operated in selective reaction monitoring mode. This work outlines the methods for both screening and identifying a wide range of small molecules, and the optimized protocol to quantify the relevant antimicrobial compounds. Such procedures could be used to support innovation and improvements for treatment processes and resource recovery in commercial food waste streams.

Another innovative use of analytical methods in foods was developed by Kurz et al. [13] to quantify the formation of disulfide bonds in protein networks. While these interactions are often critical to the functional properties of food systems, there are limited methods available for direct quantification. These authors outlined a procedure to quantify both free and total thiols, using whey protein isolate as a model system. The method employs a thiol-detecting agent (4,4’- dithiodipyridine), and quantification was conducted using RP-HPLC (RP; reverse phase). Whey proteins were evaluated both in the native state, and after thermally-induced gelation. This technique can be expected to provide more reliable results than established indirect techniques which quantify only the free thiol content. Such indirect measures of disulfide bond formation will be skewed by the production of non-disulfide thiol oxidation products. This methodology should also be applicable to other protein systems with appropriate optimization of the chromatographic parameters.

Finally, the procedure outlined by Alladio et al. [14]. is included in this collection, as it proposes a methodology for improving reproducibility and reliability of regularly performed multi-targeted GC-MS procedures. These authors present a statistical model intended to reduce the number of experiments needed for day-to-day validation. Such procedures could be valuable for improving inter- and intra-day experimental reproducibility and reliability when routine measurements are being conducted.

The studies outlined here demonstrate the broad range of challenges which are being addressed with chromatographic techniques within the areas of agriculture, food, and health/nutrition. We would like to thank each of the authors for their individual contributions; however, it is also acknowledged that this collection represents only a small sampling of the work being carried out in this area. Additionally, there will continue to be advances in instrumentation, updates to regulatory standards, and a perpetual evolution of interests in the food and health industries. As such, we expect there will continue to be many developments in the implementation of chromatographic and mass spectrometric analyses. We therefore look forward to seeing further progress in this area of analytical chemistry, and we openly invite additional contributions of such protocols for submission to MethodsX.

Andrew J. Gravelle

Editor-in-Chief | MethodsX, Food Science

*E-mail address:* agravelle@ucdavis.edu

With editorial assistance from

Arun S. Moorthy

Editorial Advisory Board member | MethodsX, Food Science
